# Key soil attributes and land-management dataset for Australian agroecosystems linking baseline and resampling surveys

**DOI:** 10.1038/s41597-026-07850-8

**Published:** 2026-07-14

**Authors:** Chiara Pasut, Christina Asanopoulos, Xueyu Zhao, Mark Farrell, Ming Li, Andrea Powell, Georgia Reed, Paul Adkins, Thomas Carter, Anna McBeath, Janine McGowan, Sheridan Morton, Steve Szarvas, Kumara Weligama, David Beutel, Hue Dang, Sara Ormond, Bonnie Armour, Glenn Brown, Doug Crawford, Mary Garrard, Frances Hoyle, Ronny Lauerwald, Raia Silvia Massad, Malcom McCaskill, Massimiliano De Antoni Migliorati, Rob Moreton, Tamara O’Keeffe, Katherine Polain, Steven Reeves, Amanda Schapel, Liz Stower, Brian Wilson, Senani Karunaratne

**Affiliations:** 1 CSIRO Agriculture & Food, Kaurna Country, Gate 4 Waite Road, Urrbrae, SA 5064 Australia; 2 CSIRO Agriculture and Food, Canberra, ACT 2601 Australia; 3 CSIRO Data61, PO Box 1130, Bentley, WA 6102 Australia; 4 Department of Primary Industries and Regions South Australia, Research Rd, Nuriootpa, SA 5355 Australia; 5 Department of Natural Resources and Environment, Hobart, Tasmania Australia; 6 Department of Energy Environment and Climate Action, 1301 Hazeldean Road, Ellinbank, VIC 3821 Australia; 7 School of Environmental and Rural Science, University of New England, Armidale, NSW 2351 Australia; 8 SoilsWest, Centre for Sustainable Farming Systems, Food Futures Institute, Murdoch University, Murdoch, WA 6150 Australia; 9 Université Paris-Saclay, INRAe/AgroParisTech, UMR EcoSys, 91120 Palaiseau, France; 10 Department of Energy Environment and Climate Action, 915 Mount Napier Road, Hamilton, VIC 3300 Australia; 11 Department of the Environment, Tourism, Science and Innovation, 41 Boggo Road, Dutton Park, QLD 4102 Australia; 12 CSIRO Agriculture and Food, St Lucia, Qld Australia

## Abstract

The Soil Organic Carbon Monitoring project provides a dataset from 308 agricultural sites distributed across Australia’s major farming regions, integrating soil attributes (biological, chemical and physical) and elemental properties with detailed temporal land-management information. Baseline soil samples were collected between 2009 and 2012 under the Soil Carbon Research Program, and the same sites were resampled between 2022 and 2025. The dataset includes laboratory measurements of soil organic carbon, total nitrogen, pH of 1:5 soil/water suspension, pH of 1:5 soil/0.01 M calcium chloride extract, electrical conductivity (EC) of 1:5 soil/water extract, whole-soil bulk density, permanganate-oxidisable carbon, and soil elemental composition measured by portable X-ray fluorescence for a wide suite of major and trace elements. Ten soil samples were collected at three depths (0 - 0.10, 0.10 - 0.20, and 0.20 - 0.30 m) on a 25 × 25 m grid, except for the rangeland sites, where a large polygon area was used as the sampling support. At some sites, cores were kept separate (not composited based on the depth supports, known as detailed sites), while at other sites, they were composited at each depth support. Particle size distribution and direct measurements of soil organic carbon fractions (particulate, humic and resistant carbon) were performed on a spatially representative subset of 300 samples, selected to capture variability in soil properties inferred through the gathered infrared spectral datasets for the whole dataset. In addition, paddock-level land-management histories spanning 2010–2023 document crop and pasture types, tillage and residue management, fertiliser application, irrigation status, and grazing. These records enable estimation of annual carbon inputs using a multi-model crop simulation ensemble. All components are spatially referenced and interoperable, allowing integration of soil properties, agriculture management practices, and positional data. This resource supports benchmarking of SOC stocks and changes, evaluation of management impacts, and development of monitoring, reporting, and verification frameworks for soil carbon and soil health in Australian agroecosystems.

## Background and summary

Healthy soils underpin crop yields and pasture biomass, directly influencing food security and farm and climate resilience. Soil organic carbon (SOC), total nitrogen, and their constituent fractions, particulate organic carbon (POC) vs. humus organic carbon (HOC) and labile vs. resistant organic carbon (char-like material ROC) along with soil pH and electrical conductivity (EC), are widely recognised as core indicators of soil quality and ecosystem health^1^. These properties reflect both the soil’s capacity to support agricultural productivity through nutrient availability, water retention, and biological activity and its role in climate regulation via long-term carbon sequestration^2^. From a climate perspective, soil is central to nature-based greenhouse gas (GHG) mitigation strategies, offering a potential pathway to offset a portion of anthropogenic emissions^3^. Recognising this dual importance, Australia has prioritised soil monitoring programs as part of its national strategy for sustainable agriculture, climate reporting, and related policy initiatives^4^.

Effective SOC monitoring requires not only measuring soil properties but also accurately estimating ancillary datasets, including carbon inputs primarily derived from data on crop yields (or biomass), pasture biomass, and residue management. These inputs play a vital role in whole-of-system analyses, as they determine the balance between carbon inputs and outputs in highly disturbed agricultural ecosystems due to different land management activities. These inputs vary significantly across farming systems due to differences in climate, soil type, and management practices, introducing substantial variation in SOC accounting and limiting the precision and accuracy of model-based projections.

To address these challenges, the Australian Soil Organic Carbon Monitoring project (SOC-M;^5^) combines soil measurements with detailed farm management data to improve estimation of carbon inputs and enable more robust assessment of management impacts on SOC dynamics. The SOC-M program builds on the Soil Carbon Research Program (SCaRP^1^), which implemented a nationally coordinated soil monitoring framework across Australia’s major agricultural regions. In this context, systematic soil sampling refers to a structured site selection approach designed to achieve broad spatial coverage across climate zones, soil types, and dominant land uses, considering key carbon inputs, decomposition and stabilisation.

SOC-M revisits a subset of SCaRP farmer paddock sites (308 of 4,180) to enable paired temporal analyses, while maintaining consistent sampling depths (0–0.10, 0.10–0.20, and 0.20–0.30 m), using a standardised field protocol^6^. At each site, ten soil cores were collected and either analysed individually or composited by depth, depending on the sampling design, to balance representativeness and analytical efficiency. The SCaRP baseline survey was conducted between 2009 and 2012 (with most sites sampled around 2012), while the SOC-M resampling took place between 2022 and 2025, providing an approximate 10–12 year interval to assess changes in soil carbon and related properties. In total, the SOC-M dataset comprises 308 agricultural sites across Australia. Complementary land management records and derived carbon input estimates span 2010–2023, supporting interpretation of observed changes. Throughout the manuscript, these periods are referred to as the baseline soil survey (2009–2012), the resampling survey (2022–2025), and the management and carbon input time series (2010–2023).

This data release delivers comprehensive measurements of SOC, including its constituent fractions following the Baldock *et al*. (2013)^1^ methodology. The dataset also includes pH of 1:5 soil/water suspension, pH of 1:5 soil/0.01 M calcium chloride extract, Electrical conductivity (EC) of 1:5 soil/water extract, whole bulk density, permanganate oxidisable carbon (POXC), and elemental composition of soils that could inform soil carbon stabilisation (Mg, Al, Si, P, S, K, Ca, Ti, V, Cr, Mn, Fe, Co, Ni, Cu, Zn, As, Se, Rb, Sr, Y, Zr, Nb, Mo, Ag, Cd, Sn, Sb, W, Hg, Pb, Bi, Th, U, LE i.e., light material with atomic number < 12) from 308 agricultural sites spanning Australia’s major farming regions. A subset of 300 samples was selected for detailed SOC fractionation analysis, namely particulate organic carbon (POC), humus organic carbon (HOC) and resistant organic carbon (ROC), based on analytical capacity and to ensure representation across major soil types and chemical composition^1^. In addition to these soil attributes, a specially designed survey gathered detailed management histories for most sites, including crop and pasture rotations, irrigation regimes (rainfed or irrigated), yields (where available), tillage and stubble practices, and nitrogen, phosphorus, and potassium fertiliser applications. These records are essential for estimating annual carbon inputs, which are a key driver of SOC dynamics.

The analysis of a comprehensive suite of soil properties supports both soil carbon accounting and broader soil condition assessment. Core variables (SOC, nitrogen, carbon fractions) quantify carbon stocks and dynamics, while ancillary properties (pH, EC, bulk density, elemental composition) provide context on soil fertility, structure, and stabilisation. Together, these enable integrated assessment of carbon change, management impacts, and soil function over time.

This harmonised, longitudinal dataset fills a critical gap by providing consistent measurements of SOC quantity and quality across diverse agricultural systems. It strengthens national greenhouse gas reporting, improves understanding of SOC dynamics, and supports the development of monitoring, reporting, and verification (MRV) frameworks at national scale.

## Methods

**Fig. 1:**
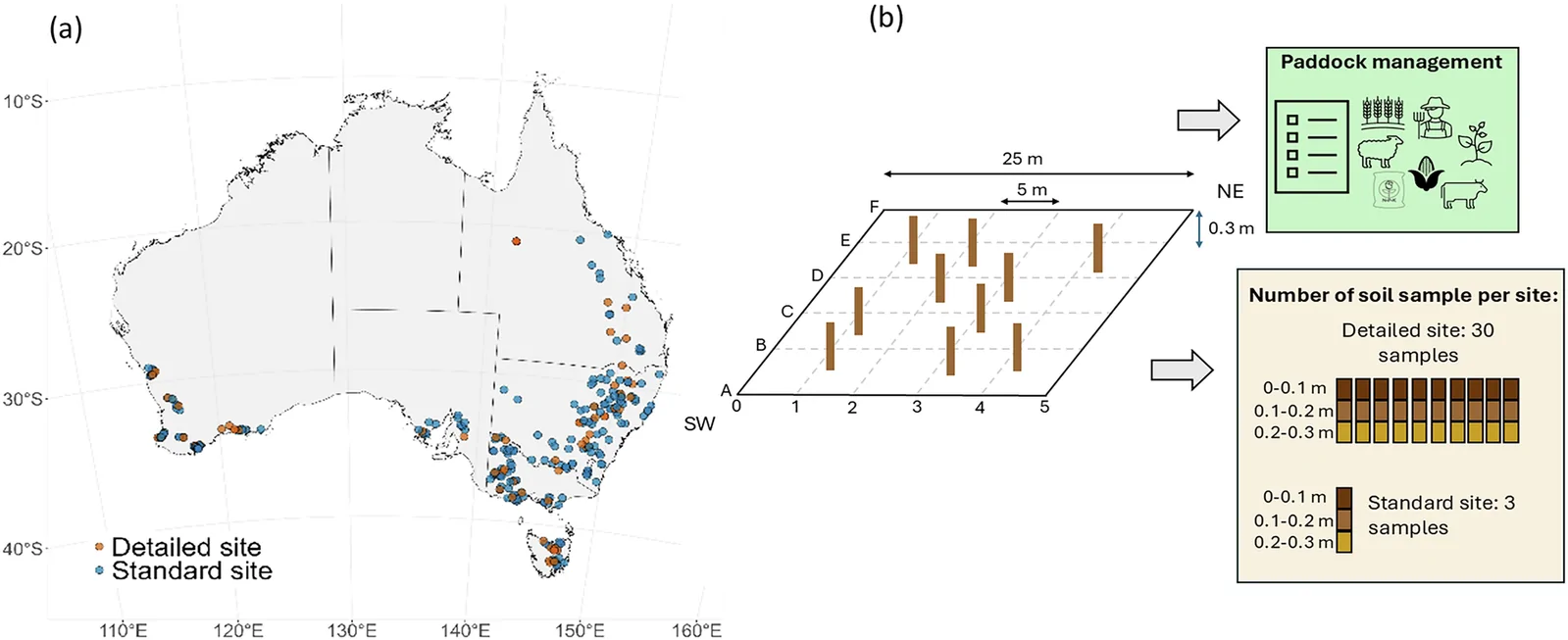
(**a**) Spatial distribution of sites sampled in the Soil Organic Carbon Monitoring (SOC-M) project. Red points indicate detailed sites (up to 30 samples per site), and blue points indicate standard sites (3 composited samples per site). (**b**) Schematic of the 25 × 25 m sampling grid used at each site. Rangeland sites had a polygon-based sampling support instead of a 25 × 25 m sampling grid.

This data release comprises four complementary components: (i) positional information collected during the field survey (croplands, pasture/grasslands and selected rangeland sites), (ii) laboratory measurements of soil key properties across the sampling support (Fig. 1), (iii) soil elemental composition, and (iv) detailed management records provided by landholders and estimated annual carbon inputs spanning the period 2010–2023. Together, these components provide a harmonised resource for examining SOC dynamics across Australian agricultural systems.

The dataset includes directly measured variables (soil properties and management records), survey collected information (farm management practices), and model-derived estimates (annual carbon inputs). These components are reported separately to clearly distinguish observational and surveyed data from model-based outputs and their associated uncertainties. In the following sections, we describe the protocols used for field data collection, laboratory analyses, compilation and gap-filling of management data, and the modelling framework used to generate carbon input estimates.

### Soil monitoring sites and sample support

The nationwide soil sampling campaign, originally conducted between 2009 and 2012 as part of the Soil Carbon Research Program (SCaRP), was revisited between 2022 and 2025 under the Soil Organic Carbon Monitoring project (SOC-M). This second-phase effort involved re-sampling 308 of the original 4,180 SCaRP sites, excluding the experimental sites (~7%, Fig. 1a). The re-sampling design was structured to capture the environmental, geographic, topographic, and soil variability with the aim to capture temporal carbon inputs, decomposition and stabilisations, considering the original site locations, while allowing flexibility to substitute alternate locations when necessary (for more details on the soil sampling design, refer to^5^).

To ensure consistent representation of agricultural management practices across uniform soil types, sampling within each paddock was conducted using a structured 25 × 25 m grid. This design ensures that the observations are representative and reduces uncertainties arising from small-scale spatial variability of soil conditions, thereby enhancing the reliability of site-level measurements. As noted, exceptions were made for Queensland rangeland sites (a total of ten sites), which used larger polygon sampling areas (varying from 60 to 100 ha, depending on the paddock).

At each selected paddock, ten sampling points were randomly located within the grid. Soil samples were collected at three depth intervals (0–0.10, 0.10–0.20, and 0.20–0.30 m) using a corer with a minimum diameter of 40 mm. At each site, field data sheets documented the coordinates of the South West and North East corners (RTK-GPS, EPSG:4326), grid orientation, and the sampling position within the 25 × 25 m grid. Surface residues and organic layers were removed prior to sampling to ensure measurements reflected the mineral soil.

Within each site, a two-tier sampling strategy was implemented. A total of 207 sites were designated as *standard sites*, where cores collected at each depth were composited to produce a single bulked sample per each depth support. These sites provide spatially integrated estimates of soil properties across the paddock. The remaining 101 sites were designated as *detailed sites*, where individual cores were retained and analysed separately to quantify within-site spatial variability. The proportion of standard and detailed sites reflects a trade-off between spatial coverage, the need to quantify within-site variability, and the constraints imposed by analytical costs. For the standard sites, compositing resulted in 3 samples being used for measurement. For detailed sites, up to 30 samples per site were analysed (10 cores × 3 depths), assuming all depths were successfully collected.

The final dataset includes information from 3,644 samples, slightly lower than the planned total of 3,651 due to occasional field constraints (e.g. incomplete core recovery or sampling barriers at depth).

### Total carbon, total organic carbon and inorganic carbon analysis

Total carbon (TC), soil organic carbon (SOC), inorganic carbon (IC), and total nitrogen contents were determined from finely ground (<2 mm) and oven-dried soil subsamples using elemental analysers (LECO TruMac, LECO Corporation, MI, USA). Calcareous soils (identified via a fizz test using 1 M HCl) were analysed for SOC after inorganic carbon (IC) was removed via acid treatment with repeated applications of 5–6% sulphurous acid (H₂SO₃). IC values < 1.0 mg C g soil^-1^ were considered below detection limits. Quality assurance involved regular use of certified standard soils, calibration checks, duplicates, and drift correction. All values were corrected to an oven-dry basis using gravimetric water at 105 °C. In the absence of the IC, the TC was considered equal to the SOC. The full laboratory pipeline is summarized in Figures S1, S2, S3, S4 in the Supplementary Information (SI). Soil carbon and related properties are reported as concentrations (%) unless otherwise stated, therefore, an equivalent soil mass (ESM) correction is not included.

### Measurable SOC fraction analysis

From the 3,644 SOC-M samples, 300 were selected for particle-size fractionation and characterisation of polyaromatic carbon, also called resistant organic carbon (ROC) using ¹³C NMR spectroscopy. This analysis yielded three measurable SOC fractions, namely POC, HOC and ROC as defined by^1^.

Two criteria were applied to exclude samples unsuitable for fractionation (SOC ≥ 5 mg C g^−1^ and ≥150 g available bulk soil). The final 300 were then selected using the Kennard–Stone algorithm^7^ from the pre-processed (i.e., baselined and trimmed) MIR spectra, ensuring representative coverage of the dataset and supporting future predictive modelling of soil properties. MIR spectra were collected using a Bruker Invenio S FT-MIR spectrometer equipped with an HTS-XT microplate module, KBr beam splitter, and liquid-nitrogen-cooled MCT detector (Bruker Optics Inc., MA, USA). For each sample, 64 scans were acquired and averaged at 4 cm^−1^ resolution over the 7,500–600 cm^−1^ wavenumber range. Absorbance spectra were used for all analyses, with data interpretation focused on the 6,000–600 cm^−1^ region. Instrumental baseline variation was minimised by recording a background spectrum prior to each sample run.

For each selected sample, 10 g of <2 mm soil was dispersed overnight in sodium hexametaphosphate (5 g L^−1^) and wet-sieved at 50 µm using an automated vibratory system. This procedure separated two size fractions: coarse (2000-50 µm) and fine fractions (<50 µm). Two size fractions were collected into pre-weighed bottles, freeze-dried, and weighed to determine mass recoveries, which were typically >98%. Carbon mass recoveries between 85–115% were accepted; samples outside this range were reanalysed. Soil organic carbon concentrations in each fraction were measured by dry combustion analytical approach. For calcareous samples, an acid pre-treatment was applied to remove carbonates^6^. Coarse fractions were further purified to remove mineral material (sand) through flotation and filtration. Fine fractions were accumulated by additional wet sieving of up to 40 g of <2 mm soil (depending on expected OC content), followed by freeze-drying. The fine material then underwent sequential 2% HF treatments^1^ to reduce mineral interference (iron, manganese, and other paramagnetics) and enrich the organic component when running the ^13^C NMR spectroscopic analysis. The overall workflow for fractionation and carbon recovery is summarised in Figure S4 (SI).

Solid-state ¹³C NMR spectra were obtained to characterise the char-like material present in both the coarse and fine fractions. Samples were analysed using a Bruker 400 MHz Avance NEO spectrometer (9.4 T magnet; 100 MHz ¹³C frequency) with cross-polarisation (CP) acquisition (1 ms contact time, 1 s recycle delay) and a minimum of 10,000 scans per sample (up to 50,000 for soils with low OC). For SOC fraction analysis, CP-NMR inherently underestimates the ROC pool while overestimating the POC and HOC fractions, due to its sensitivity to the observation of aromatic carbon structures. To correct for this bias, 14 samples of the 300 were additionally analysed under direct polarisation (DP)Magic Angle Spinning (MAS) conditions, which detect only ¹³C nuclei. These 14 samples were combined with SCaRP original calibration set (n = 39) to improve the representation of SOCM samples in the applied correction factor^6^. Although DP experiments require 24 hours or more per sample (compared with 4–6 hours for CP), they provide an unbiased reference for quantifying carbon functional groups. By analysing these samples with both CP and DP, quantification of polyaromatic carbon was completed, allowing for correction of the CP-derived estimates^1^. This correction factor was applied to all SOC-M CP-NMR data, and used to derive POC, HOC, and ROC concentrations for whole soils based on the combined results of the coarse and fine fractions.

### Measurement of pH (water), pH (CaCl_2_), and electrical conductivity (EC)

Approximately 5 g of <2mm air-dried soil was transferred into a 50 mL centrifuge tube and combined with 25 mL of deionised water. The mixture was shaken for 1 hour on an end-over-end shaker to produce a soil suspension. Electrical conductivity was determined on this suspension using a conductivity meter calibrated against appropriate standards. Soil pH in 1:5 of soil/water suspension (pH) was measured on the same suspension with a calibrated pH electrode under continuous stirring. Subsequently, 1.25 mL of 0.21 M CaCl₂ solution was added to the suspension, mixed thoroughly, and left to stand for 30 min. Soil pH in CaCl₂ (pH_CaCl2_) was then determined by placing electrodes in the unstirred supernatant and recording the value once a stable reading was obtained.

### Measurement of ancillary soil physical properties – whole soil bulk density and particle size analysis

All soil samples were analysed for whole-soil bulk density following the procedures described in the Supplement to the 2021 Soil Organic Carbon Measurement and Modelling Methodology (2021 soil carbon method, Australian Government 2021), and details are provided in^6^.

Clay, silt, and sand contents for the 300 samples selected for physical SOC fractionation were determined using the Micropipette Method (MPM–Clay), following the procedures of^8^ and^9^. Briefly, approximately 10 g of air-dried (<2 mm) soil was dispersed in a 5% sodium hexametaphosphate solution and deionised water, and thoroughly mixed to achieve complete particle dispersion. The suspension was then transferred to a 1 L sedimentation cylinder, diluted to volume, and allowed to settle under still conditions, usually 24 hours. An aliquot was withdrawn from a fixed depth at a specified time using a calibrated micropipette, following Stokes’ Law with temperature corrections applied. The aliquot was dried to constant weight, and the clay percentage was calculated from the resulting residue mass. Consistent pipetting depth, timing, and sample handling were maintained throughout the analysis to ensure reproducibility.

### Soil elemental composition

Approximately 30 g of <2 mm soil was placed into 50 µm plastic bags for pXRF analysis. Measurements were performed using an Olympus Vanta analyser equipped with a Rhodium (Rh) anode 50 kV X-ray tube and a large-area Silicon Drift Detector (SDD). Elemental concentrations were estimated using the factory-installed GEOCHEM mode calibration, which reports results in ppm. The GEOCHEM configuration employs a dual-beam system (50 kV and 10 kV) to quantify a wide suite of elements, including Mg, Al, Si, K, Ca, S, P, Ti, V, Cr, Mn, Fe, Co, Ni, Cu, W, Zn, Hg, As, Pb, Bi, Se, Th, U, Rb, Sr, Y, Zr, Nb, Mo, Ag, Cd, Sn, and Sb, each with element-specific limits of detection (LODs). To enable detection of lighter elements such as Mg (LOD ~2500 ppm), Al (LOD ~350 ppm), and Si (LOD ~200 ppm), longer count rates are applied. In addition to processed elemental concentrations, the raw pXRF spectra from each beam setting are also available. Further details of the analytical protocol are provided in^10^.

### Permanganate oxidizable carbon - POXC

Permanganate oxidizable carbon was determined following the Ohio State University Soil Fertility Lab protocol^11^. Briefly, 2.5 g of air-dried soil (<2 mm) was reacted with 0.2 M KMnO₄ solution in the presence of CaCl₂ to maintain ionic strength. Samples were shaken for 2 minutes at 180 rpm, allowed to settle for 10 minutes, and a 1 mL aliquot of the supernatant was diluted in 49 mL of Milli-Q water. Absorbance of the diluted solution was measured at 550 nm using a microplate reader, alongside a set of KMnO₄ standards prepared from the stock solution. Concentrations were calculated based on the difference in absorbance between standards and samples, expressed on an oven-dry basis (mg C kg^−1^ soil). Quality control included duplicate samples and daily preparation of working standards to ensure consistency across runs.

### Paddock management data

The SOC-M and SCaRP projects’ majority of the sites detailed land management records of the decades before sampling. Information included crop types, yields, tillage methods, stubble management, irrigation, fertiliser applications, and grazing strategies. These records enabled the reconstruction of annual management histories for 308 agricultural sites across Australia resampled under the SOC-M project.

To enable comparison across sites and survey periods i.e. SCaRP and SOC-M, management information was standardised into broader categories. Tillage practices were grouped into four classes (Table S1): conventional (>2 working passes per season), reduced (one additional pass beyond sowing), minimum/no-till (sowing only), and deep ripping (strategic soil amelioration). Stubble management was classified into five categories: retained on the surface, retained but incorporated, grazed, baled/removed, or burnt. Gap-filling rules were applied to maintain continuity, whereby perennial or mixed pasture years with missing information were imputed using defaults of no tillage, residue grazed, and rainfed management. Missing cropland years were excluded because crop rotations could not be reliably reconstructed.

### Crop yield and pasture biomass data

Annual carbon inputs were estimated from crop yields or pasture biomass, adjusted for stubble management. However, yield data were not consistently available across all sites. To address this, resampled sites were classified into two groups: data-rich sites, where crop type, yield or biomass, grazing, tillage, and stubble management were fully recorded (approximately 50% of sites); and data-poor sites, where management sequences were available but yield or biomass records were missing (approximately 50% of sites, particularly pasture-dominated systems). Carbon input time series were generated for both categories (more details in Section 2.14). Hence, Carbon input estimates presented here are model-derived variables and should be interpreted as approximations of system-level inputs rather than precise site-level measurements. These components are reported separately to distinguish observational data from model-based outputs and their associated uncertainties. Both observed and model-derived carbon inputs are provided in the dataset (Table 4; *C_input_Observed_year* and *C_input_modAveg_year*).

Given the high uncertainty in key system variables such as crop types, sowing dates, fertiliser application timing, and cultivar selection, which propagate into carbon input estimates, a multi-model ensemble framework was used. Three crop models were applied: APSIM-NextGen^12^, CERES-EGC^13^, and the CSIRO crop model^14,15^. Because no single model can represent all relevant crop types or pasture systems across Australia, the ensemble approach ensures broader system coverage and reduces structural bias. From 2010 to 2023, farm-level yields were simulated and assessed against ~1,200 yield and biomass observations supplied by landholders, providing both validation and a measure of predictive uncertainty.

Model outputs were independently validated and then combined into an ensemble by averaging predictions. This ensemble approach was adopted to reduce model-specific biases and improve the robustness of carbon input estimates^16^. At a subset of data-rich sites, selected crop parameters in the CERES-EGC model were fine-tuned to reflect Australian production systems better; however, formal calibration was not the primary objective of the work presented here (Figure S5).

### Model descriptions

#### APSIM-NextGen

APSIM, including its update APSIM-NextGen, is a widely used crop systems model in Australia and elsewhere, designed to simulate agricultural management and crop rotations under diverse environmental conditions^17^. For this study, simulations were conducted for the period 2010–2023 using site-specific inputs, including crop type, sowing and harvest dates, cultivar, planting density, fertiliser application, tillage, stubble management, and irrigation regime. The soil profile was modelled to a depth of 0.5 m, divided into four layers (0.1, 0.1, 0.1, and 0.2 m thick), with the first three layers aligned to observed sampling depths and the fourth supporting free drainage and nutrient redistribution, particularly under deep ripping tillage management. For irrigated systems, a water-deficit-based rule was applied, triggering irrigation when soil moisture in the top 0.3 m fell below 90% of plant-available water. A minimum interval of seven days was enforced between irrigation events, and irrigation was restricted within two days of sowing. Fertiliser rates were derived from landholder records; where timing data were unavailable, fertiliser was assumed to be applied entirely at sowing for rates ≤150 kg N ha^−1^, and split into 60% at sowing and 40% six weeks later for higher rates. Tillage and stubble operations were parameterised to reflect reported practices: conventional tillage to 250 mm, reduced tillage to 150 mm, deep ripping to 600 mm, and with 90% residue removal in cases of grazing, baling, or burning.

#### CSIRO yield model

This water-balance model simulates daily crop and pasture growth to calculate annual crop yields and monthly pasture biomass using rainfall, evapotranspiration, and crop-specific parameters. It uses general fixed sowing days specific to crop or pasture species based on the geographic region, on available survey datasets generated and saved in a background database, which is used as a model input, which does not require information on sowing dates, fertiliser inputs, or cultivar, making it suitable for large-scale applications. The model underpins Australia’s National Greenhouse Gas Inventory land sector accounting system, FullCAM framework^14^. Outputs are reported at the Statistical Area Level 2, which is considered an administrative region, a polygon-based unit in Australia (ASGS 2016).

#### CERES-EGC

CERES-EGC is a crop production model similar to APSIM. The CERES-EGC (Crop Environment Resource Synthesis - Environnement et Grandes Cultures) model is based on the CERES family of models developed in the United States by^18^. These models are developed and distributed within the international DSSAT (Decision Support System for Agrotechnology Transfer) network, which allows for its validation under various pedoclimatic (soil and climate) and agricultural conditions^19^. It models carbon and nitrogen dynamics, heat transfer, and water exchange between soil, plants, and the atmosphere. Inputs include weather data (e.g. temperature, rainfall, radiation), soil properties, crop characteristics, and management practices like tillage, irrigation, and fertilisation. The model incorporates the NCSOIL module^20^ to simulate soil organic matter decomposition and nitrogen transformations, and the Nitrous Oxide Emissions (NOE) module^21^ to estimate N₂O emissions. It supports a range of crops and accounts for plant growth, nitrogen uptake, and losses such as volatilisation, constrained by environmental conditions and crop-specific traits. The simulation setup followed the standard set of rules as for APSIM-NexGen.

### Data pre-processing

Both APSIM-NextGen and CERES-EGC are detailed, process-based crop models that require extensive input information, including crop rotations, cultivar identity, plant density, and sowing dates. In contrast, the CSIRO model relies primarily on climatic variables, soil physical properties, and generalised crop parameters, resulting in substantially lower data requirements. Cultivar-specific traits (e.g., phenology, population, row spacing) were assigned according to crop type (Table S2). Because consistent cultivar metadata were not available across the full monitoring period, we assumed a constant cultivar for each crop, thereby limiting unnecessary sources of uncertainty and allowing us to focus on management and environmental drivers of yield and carbon inputs.

Sowing dates were handled differently across models. CSIRO’s yield model does not require explicit sowing-date inputs, as these are internally encoded using location-specific rules. APSIM-NextGen and CERES-EGC, however, require sowing dates, which were inconsistently reported in the survey. To address this limitation, we used the global crop calendar dataset via public repositories^22^, refined using rainfall thresholds (25 mm over 7 days) to identify sowing events^23^, and applied mean sowing dates when thresholds were not met.

All models were driven by the same set of environmental data, except the CSIRO yield model. Daily climate variables (rainfall, temperature, evaporation, radiation) were obtained from the SILO 5-km grid^24^ (https://www.longpaddock.qld.gov.au/silo/). CSIRO yield model used 1 km grided climate variables. Soil physical and chemical properties (e.g., texture, hydraulic conductivity) were extracted from the Soil and Landscape Grid of Australia^25^ (https://www.clw.csiro.au/aclep/soilandlandscapegrid/) via API, and initial soil water conditions were sourced from the Australian Bureau of Meteorology^26^ (http://www.bom.gov.au/water/landscape/) for the two process-based models.

Details of model assumptions, cultivar settings, input requirements, CERES-EGC calibration performance, and crop-specific validation are provided in the Supplementary Information (Tables S2–S4; Figures S5–S6).

### From yield to carbon input

Using both on-farm data supplied through surveys and model-generated estimates of yield (for crops) and biomass (for pastures) across both data-rich and data-poor sites noted earlier—annual carbon inputs were calculated using Eqs. (1–3). These calculations accounted for crop-specific yield components, allocation of total plant dry matter, and the carbon fraction of each crop type (Table S5 from^27^). The final dataset reports the observed (if provided by the landholder), minimum, maximum, and mean carbon input values derived from the ensemble.

The effect of stubble management was represented using a *retaining factor*, defined as the proportion of above-ground carbon that remains in the system after harvest. Grazed stubble was assigned a retaining factor of 0.6, reflecting the assumption that approximately 40% of above-ground carbon is removed through livestock products (e.g., meat, wool). Stubble that was baled or otherwise removed from the field was assigned a retaining factor of 0.1, consistent with ~90% of carbon being exported off-site. Burnt stubble was likewise assigned a retaining factor of 0.1, assuming that most above-ground carbon is lost via combustion (primarily as CO₂ and CH₄). In contrast, retained stubble was represented with a retaining factor of 1.0, indicating complete retention of above-ground carbon within the field.

If the yield component = grain:

1 $${{\rm{C}}}_{{\rm{input}}}=\left({Yield}\times \frac{{allo}{c}_{{Stlk}}}{{allo}{c}_{{Gbfr}}}\times {CFra}{c}_{{Stlk}}+{Yield}\times \frac{{allo}{c}_{{Leaf}}}{{allo}{c}_{{Gbfr}}}\times {CFra}{c}_{{Leaf}}\,\times {retaining}\,{factor}+{Yield}\times \frac{{allo}{c}_{{Cort}}}{{allo}{c}_{{Gbfr}}}\times {CFra}{c}_{{Cort}}+{Yield}\times \frac{{alloc}{\rm{\_}}{Firt}}{{alloc}{\rm{\_}}{Gbfr}}\times {CFra}{c}_{{Frit}}\right)$$

If the yield component = leaves:

2 $${{\rm{C}}}_{{\rm{input}}}=\left({Yield}\times {CFra}{c}_{{Leaf}}\times {retaining}\,{factor}+{Yield}\times \frac{{allo}{c}_{{Firt}}}{{allo}{c}_{{Leaf}}}\times {CFra}{c}_{{Firt}}\right)$$

If the yield component = stalk:

3 $${{\rm{C}}}_{{\rm{input}}}=\left({Yield}\times \frac{{allo}{c}_{{Leaf}}}{{allo}{c}_{{Stlk}}}\times {CFra}{c}_{{Leaf}}+{Yield}\times \frac{{allo}{c}_{{Firt}}}{{allo}{c}_{{Stlk}}}\times {CFra}{c}_{{Firt}}\right)$$

where *Yield* is the annual crop yield (Mg ha^−1^); alloc_* denotes the proportion of total plant dry matter allocated to each plant component (*Gbfr, Leaf, Stlk, Firt, Cort* for grain, leaves, stalks, fine roots, and coarse roots, respectively); and CFrac_* is the carbon fraction of each component, expressed as the proportion of dry matter that is carbon. Carbon-input estimates (C_input_) are reported in Mg C ha^−1^.

### Data quality control and assurance, and model performance metrics

For quality control and assurance of the analytical dataset we applied Bland–Altman analysis between the SCaRP database (used as baseline) and the SOC-M database^28^. This method plots the difference between paired measurements against their mean, allowing assessment of systematic bias (mean difference) and limits of agreement (±1.96 standard deviations). Unlike correlation or regression approaches, Bland–Altman analysis explicitly quantifies both the direction and magnitude of disagreement, providing insight into whether model estimates systematically over- or under-predict observed values across the measurement range.

The performance of the yield model was evaluated using standard statistical indicators, including the Root Mean Square Error (RMSE), relative bias, and Lin’s Concordance Correlation Coefficient (LCCC). These metrics assess different aspects of model accuracy and agreement with observed data. Temporal trends were assessed using the Mann-Kendall test at sites with more than four observations. The consistency of these trends was evaluated by examining their alignment with the 45-degree line of perfect agreement.

### Ethical approval

The survey campaign and research design were both approved separately by the Commonwealth Scientific and Industrial Research Organisation (CSIRO) Human Research Ethics Committee (Ethics Clearance 032/22 SOCM) and the Murdoch University Human Research Ethics Committee.

## Data Record

The SOC-M database is organised into four main components: field information, analytical data, elemental composition, and farm management data.

### Field information file

This file provides site-level metadata, including grid coordinates (South West and North East corners), sampling dates, and the location of individual cores within the 25 × 25 m sampling grid. For rangeland sites, a dedicated sub-file is provided, as the spatial support is polygon-based; in this case, the latitude and longitude of each core are reported individually. A detailed description of each variable is provided in Table 1.

**Table 1:** Description of the field information file, for both croplands and rangelands.

Variable	Description
SCaRP_ID	ID of the site
Date	when the sampling took place
Orientation	Orientation of the 25 × 25 m sampling grid within the paddock (degrees from north), used to define grid alignment during sampling.
SW_Lat	latitude of the South West corner (EPSG:4326)
SW_Lon	longitude of the South West corner (EPSG:4326)
NE_Lat	latitude of the North East corner (EPSG:4326)
NE_Lon	longitude of the North East corner (EPSG:4326)
CoreID_j	location of sampling within the grid reference

### Analytical file

This file contains measurements of selected soil physical and chemical properties. Each row corresponds to a single soil sample (standard or detailed) at a given depth interval. A detailed description of each variable is provided in Table 2.

**Table 2:** Description of the analytical file.

Variable	Unit	Description
Site		Standard; Detailed
SOCM_ID		Unique sample identifier: Standard site: SCaRPID + LowerDepth (e.g., SA00005_10, SA00005_20, SA00005_30; Detailed site: SCaRPID + LowerDepth + Grid refrence (e.g., SA000025_10_2B, SA000025_10_4F), where the grid reference (e.g. “2B”) indicates the sampling location within the 25 × 25 m grid (row–column position, as per Fig. 1).
SCaRP_ID		General label for the paddock/site
Sample_ID		unique sample ID, necessary to merge elemental composition
n_cores	integer	number of cores bulked together
u_depth	cm	upper depth of the core
l_depth	cm	lower depth of the core
Carbonate	logical	Indicator of inorganic carbon presence (0 = absent, 1 = present), used to identify samples requiring carbonate removal for accurate SOC determination.
TC_ODSoil	mg C g soil^−1^	Total carbon concentration, oven dried corrected
OC_ODSoil.x	mg C g soil^−1^	Soil organic carbon concentration, oven dried corrected
IC_ODSoil	mg C g soil^−1^	Inorganic carbon concentration, oven dried corrected
TN_ODSoil	mg N g soil^−1^	Total nitrogen concentration, oven dried corrected
BD	g cm^−3^	bulk density
Pgrav		fraction of gravel (>2 mm)
OC_Stock	mg C ha^−1^	organic carbon stock
OC	mg C g soil^−1^	Soil organic carbon concentration
POC	mg C g soil^−1^	Particulate organic carbon measured via ^13^C NMR
HOC	mg C g soil^−1^	humic organic carbon measured via ^13^C NMR
ROC	mg C g soil^−1^	Resistant organic carbon measured via ^13^C NMR
Sum	mg C g soil^−1^	sum of POC, HOC and ROC measured via size fractionation and ^13^C NMR
Recovery *OC < 2 mm	%	Carbon recoveries, calculated as a percentage of the sum of the three derived fractions out of the SOC in <2 mm fraction determined by dry combustion
EC	µS cm^−1^	Electrical conductivity
pH(water)	—	pH in water
pH(CaCl2)	—	pH in calcium chloride
s_id	Integer	Site ID label for Soil National Archive
o_id	Integer	observation ID for Site (1 for Standard sites (samples taken from bulked cores), 1 to 10 for Detailed sites (samples taken from single cores))
h_no	Integer	horizon (layer) ID for the Observation (1 = 0-0.10 m, 2 = 0.10-0.20 m, 3 = 0.20-0.30 m)
samp_no	Integer	sample number for the Horizon (1 - most sites have only one sample per observation-horizon)
jar_no	integer	jar number for the sample stored in the National Soil Archive
spec_id	Integer	Unique numeric ANSA specimen ID generated for the Australian National Soil Archive
poxc_odsoil	mg C kg^−1^ soil	Permanganate oxidizable carbon concentration, oven dried, corrected
Clay	%	clay percentage from Micropipette Method (MPM–Clay) following the procedure described by^8^ and^9^
Sand	%	sand percentage from Micropipette Method (MPM–sand) following the procedure described by^8^ and^9^
Silt	%	silt percentage from Micropipette Method (MPM–silt) following the procedure described by^8^ and^9^

### Elemental composition file

This file includes all the elemental composition measures using pXRF. Each row corresponds to a single soil sample (standard or detailed) at a given depth interval. A detailed description of each variable is provided in Table 3.

**Table 3:** Description of the soil elemental composition file.

Variable	Units	Description
Sample_ID		sample unique ID, necessary to merge the analytical values
Mode		two beams = Geochem(II) or one beam = Geochem
XX_Concentration	ppm	Concentration of Mg, Al, Si, P, S, K, Ca, Ti, V, Cr, Mn, Fe, Co, Ni, Cu, Zn, As, Se, Rb, Sr, Y, Zr, Nb, Mo, Ag, Cd, Sn, Sb, W, Hg, Pb, Bi, Th, U, LE (light material – atomic number < 12)

### Farm management file

This file documents annual paddock-level management information, including crop or pasture type, tillage, stubble practice, irrigation regime, and fertiliser application rates. Each row represents a paddock, with management records linked to the analytical data through a common site identifier (*SCaRP_ID*). Columns represent management variables by year, with more details in Table 4.

**Table 4:** Description of the farm management file.

Variable	Description and options
SCaRP_ID	General label for the paddock/site
State	New South Wales (NSW), Queensland (Qld), Tasmania (Tas), Victoria (Vic), South Australia (SA), Western Australia (WA)
Latitude	South west corner of the plot
Longitude	South west corner of the plot
ManageClass_2012	General land use during the SCaRP project (Cropland, Pasture)
ManageClass_2023	General land use during the SOCM project (Cropland, Pasture)
Crop_year	Crop or pasture type from year 2000 to 2023 (one value each year). Wheat; Oats; Sorghum; Barley; Chickpea; Mungbean; Cotton; Canola; Pigeon Pea; Maize; Cowpeas; Sunflower; Fababean; Lucerne; Lupins; Lentils; Triticale; Millet; Soybean; Oilseed; Sugarcane; Unknown - previous landowner management; Cereal; grain legume; non-root vegetable; poppies; oilseed; no crop; No Pasture; Annual pasture-grass dominant; Annual pasture-legumes dominant; Annual pasture-mixed grass/legume; Perennial pasture-grass dominant; Perennial pasture-mixed grass/legume; Mixed annual/perennial-grass dominant; Mixed annual/perennial-legume dominant; Mixed annual/perennial-legume dominant; Mixed annual/perennial-mixed grass/legume; Native; Unimproved pasture (unmanaged)
Till_year	zero till-only sowing; 1 working + sowing; conventional- > 2 working; Deep ripping
Stubble_year	residue retained on surface; residue baled and removed; residue grazed; residue retained by worked in; residue burnt
NFert_year	Fertilizer application rates in kg N ha^−1^
PFert_year	Fertilizer application rates in kg P ha^−1^
KFert_year	Fertilizer application rates in kg K ha^−1^
Irr_year	Rainfed (0), Irrigated (1)
Yield_year	crop yield or pasture biomass provided by the landholder in Mg ha^−1^
C_input_ModAveg_year	average carbon input calculated from the model ensemble (Mg C ha^−1^)
C_input_Observed_year	carbon input calculated from the landholder provided yield (Mg C ha^−1^)
Max_C_input_year	Maximum carbon input calculated from the model ensemble (Mg C ha^−1^)
Min_C_input_year	Minimum carbon input calculated from the model ensemble (Mg C ha^−1^)

The four components are designed to be interoperable: the analytical and elemental composition data can be linked using the *Sample_ID*, while the *SCaRP_ID* allows integration with farm management and field information. This structure enables users to combine soil properties, management histories, and spatial context into a unified dataset for analysis.

## Link secondary data

The SOC-M dataset can be linked to baseline sampling, the Soil Carbon Research Program (SCaRP) via the common *SCaRP_ID*. When combined with the publicly available SCaRP dataset, accessible through the CSIRO Data Access Portal^29^, this linkage provides a unique resource: 308 agricultural sites with two independent soil carbon measurements and fractions approximately a decade apart, with publicly available positional information. This combined dataset allows researchers to investigate SOC change over time while situating those changes within a detailed framework of climate, management, and spatial information. The crop types in SOC-M are more detailed, but for comparison we can group into broad categories, for instance: cereals (wheat, barley, sorghum), pulses (chickpea, mungbean, lupin, lentil, soybean, etc.), oilseeds (canola, sunflower), cotton, maize, poppies, hay (oats, lucerne, triticale), pastures (annual or perennial, grass- or legume-dominant), and fallow (no crop present).

## Archival of physical samples

All 3,644 soil specimens collected by SOC-M are archived within the CSIRO Australian National Soil Archive (ANSA) in Canberra, a long-term repository that ensures secure storage and accessibility for future research. Samples are only accepted into the archive once their associated data has been validated and ingested into the NatSoil database, which is publicly accessible via the CSIRO Data Access Portal (https://doi.org/10.25919/c4br-0r30). Each physical soil sample can be uniquely identified using the identifiers s_id, o_id, h_id, samp_id, jar_id, and spec_id (see Table 2), which enable tracking, retrieval from the Australian National Soil Archive, and further analysis of individual samples.

## Technical Validation

### Paddock location re-sample location accuracy check

To ensure consistency and comparability with the previous sampling, we verified that the southwest corner of each grid was within ± 10 m of the original location. Twelve sites were relocated due to the construction of new roads, changes in paddock boundaries, or errors in the original GPS records (summarised in Table 5). In addition, five sites reported only the southwest corner and not the northeast corner, due to issues with the recorded GPS coordinates and support grids exceeding 25 m (DECCW_NA_029, DECCW_SR_03, MC_053, NSW_6_6, NSW_95).

**Table 5:** Summary of the sites where the grids had moved more than 7 m from the original sampling location.

SCaRP_ID	Latitute_SW_SCaRP	Latitute_SW_SCaRP	Latitute_SW_SOCM	Latitute_SW_SOCM	Distance (m)	Note
NSW_10b_7	−30.00729	149.9714	−30.00747	149.9713	20.4	Road on the original SW, grid moved 15 m South
NSW_264	−32.1763	149.5604	−32.1763	149.5601	28.7	Possible error from the GPS
NSW_374	−33.95372	148.3901	−33.95368	148.3903	25.1	grid moved 20 m to be inside the paddock
NSW_4a_24	−31.3278	149.4713	−31.3269	149.4713	100	Site located 100 m N, probably SCaRP locations incorrect (they point to a flooded area)
NSW_6_6	−31.43599	150.3736	−31.44507	150.3762	1,000	Incorrect SCaRP location, landholder identified correct location
SCWA_0442	−29.5248	115.304	−29.52481	115.303	99.8	Error in GPS data in SCaRP
TAS_0095	−42.45114	146.7257	146.7257	146.7256	20.3	grid moved 20 m S to represent the paddock
TAS_0140	−42.65496	147.4581	−42.6548	147.4581	17.0	grid moved 17 m N to represent the paddock
TAS_0176	−41.13097	147.4503	−41.13112	147.4503	16.1	grid moved 16 m S to represent the paddock
TAS_0202	−42.17688	147.5074	−42.17688	147.5076	12.0	grid moved 12 m E to represent the paddock
TAS_0311	−42.46634	147.1637	−42.46634	147.1635	15.9	grid moved 16 m W to represent the paddock
Vic_0017	−38.13245	143.3058	−38.13247	143.3055	26.5	GPS error in SCaRP

### Bulk density validation check

**Fig. 2:**
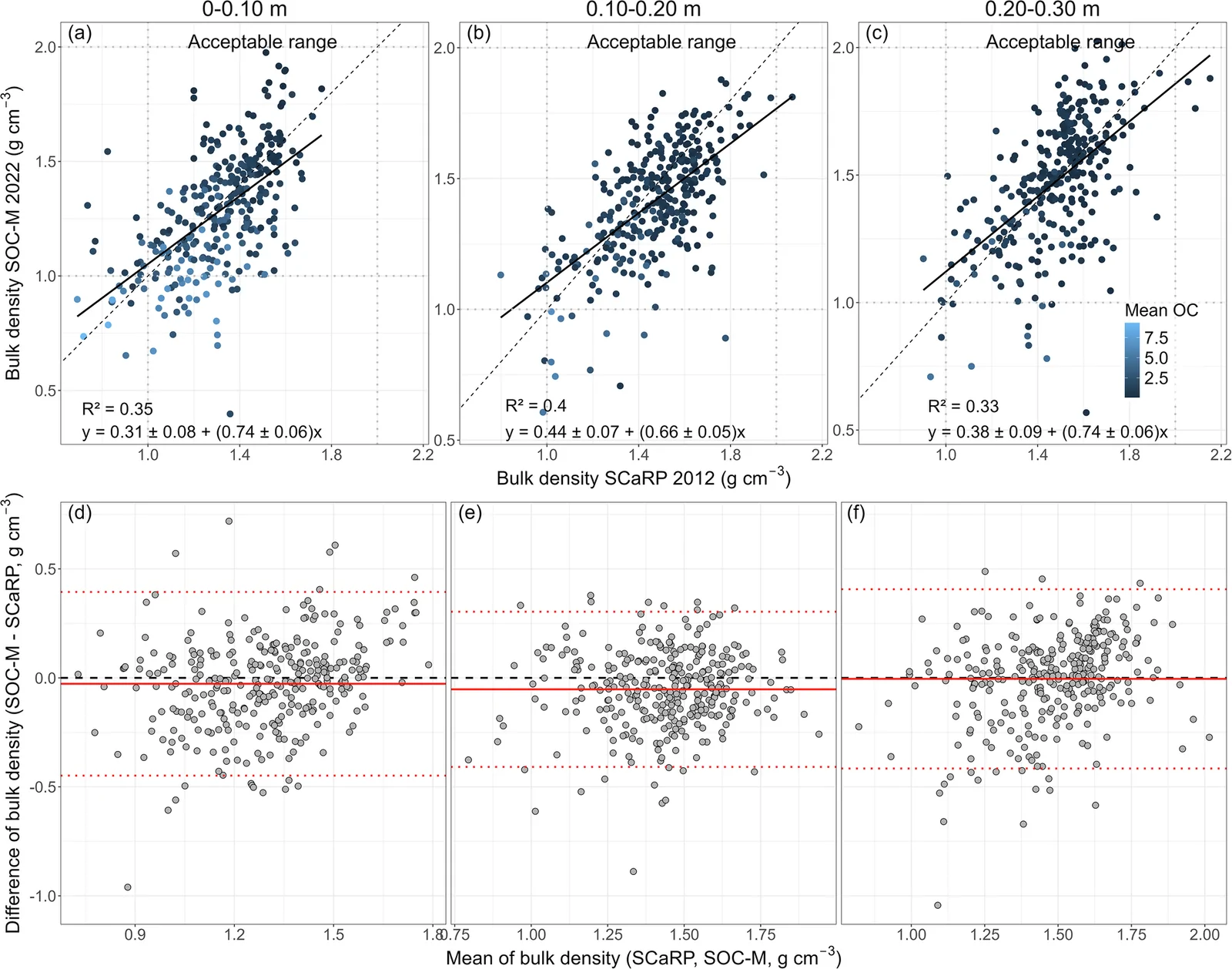
Variation of measured whole soil bulk density between SCaRP and SOC-M projects. The lighter the color is, the higher the soil organic carbon content (in %). Panels in the first row shows the scatter plots, while the second-row panel shows the Blant- Altman plot for the deviation and bias. The equation represents the linear fit, including the standard errors around the slope and interception.

Bulk density is expected to vary across space and time due to differences in soil structure, land management, and environmental conditions; therefore, comparisons between surveys are interpreted as indicators of consistency rather than direct measures of bias. Hence, to assess the consistency and plausibility of bulk density measurements between the two sampling campaigns, we compared site- and depth-matched observations, assessed whether values fell within physically acceptable ranges, and examined whether low bulk density values coincided with high soil carbon content. Scatterplots showed a moderate correlation between surveys (R² = 0.33–0.40 across depth intervals), with regression slopes close to the 1:1 line, indicating broadly comparable estimates between the two programs (Fig. 2). The majority of bulk density values (~95%) fell within the expected range for agricultural mineral soils of 1–2 g cm^−3^. In the top 0–0.1 m, some unusually low values were observed, but these coincided with soils containing very high carbon concentrations (>7%). Bland–Altman analysis further confirmed that mean differences were close to zero across all depths, indicating no strong systematic offset, although variability reflects expected spatial and temporal differences in bulk density.

### Soil carbon content

**Fig. 3:**
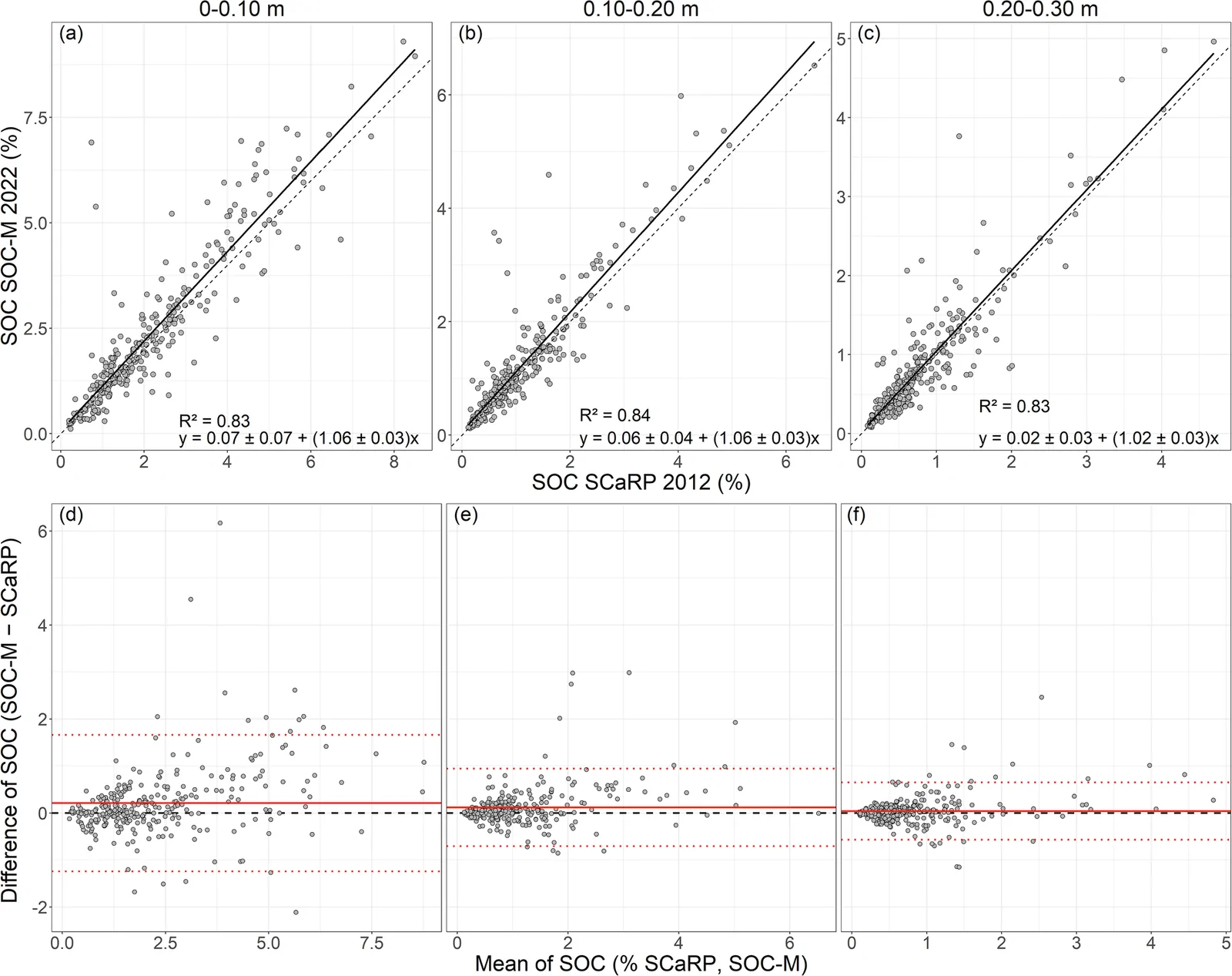
Variation of measured soil organic carbon content between SCaRP and SOC-M projects. Panels in the first row shows the scatter plots, while the second-row panel shows the Blant- Altman plot for the deviation and bias. The equation represents the linear fit, including the standard errors around the slope and interception.

To validate consistency between the two temporal surveys, paired site- and depth-matched SOC samples (0–0.30 m) were compared across SCaRP and SOC-M. Scatterplots showed strong correlations (R² = 0.83–0.84 across depth intervals, Fig. 3), with regression slopes closely aligned to the 1:1 line, indicating high agreement. Bland–Altman analysis further demonstrated that mean differences were close to zero across depths, with no systematic bias and the vast majority of samples within narrow limits of agreement.

### Carbon to nitrogen ratio

**Fig. 4:**
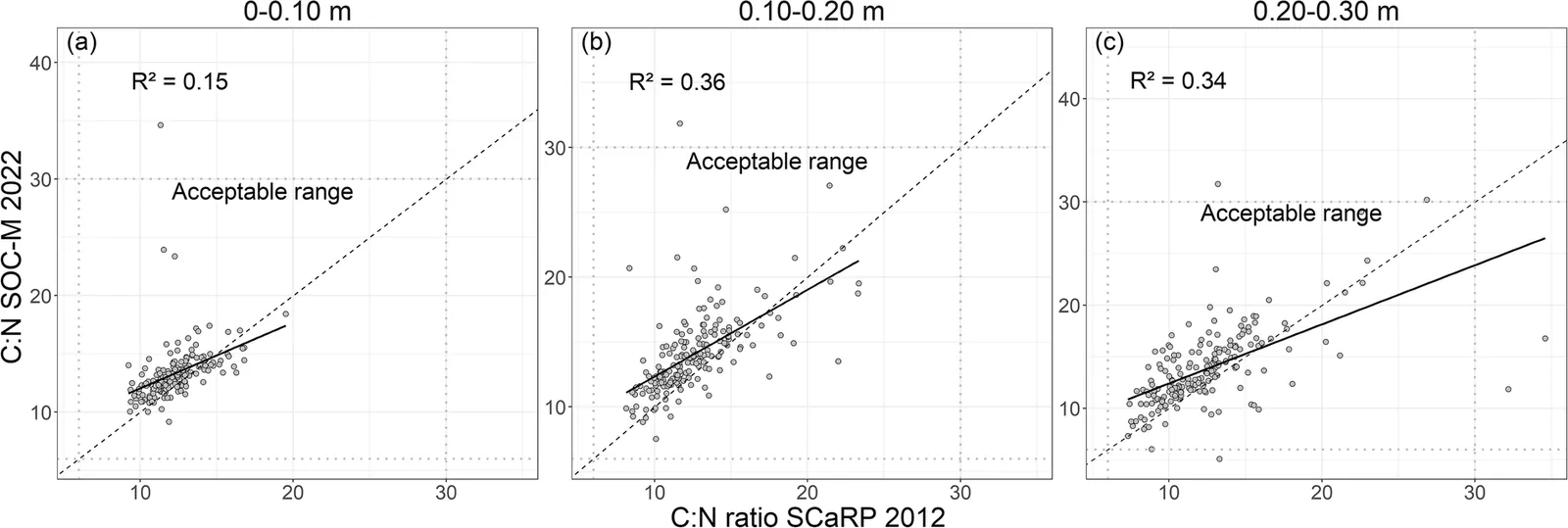
Variation of the measured total carbon to total nitrogen ratio between SCaRP and SOC-M projects.

C:N ratios (calculated as ratio between total carbon and total nitrogen) are expected to vary over time due to changes in management, plant inputs, and seasonal conditions; therefore, comparisons between surveys are interpreted as indicators of consistency and plausibility rather than preservation of a fixed relationship. Thus, we compared C:N ratio across depths measurements between SCaRP (2012) and SOC-M (2022). Correlations between surveys were relatively weaker than for bulk density or SOC concentrations (R² = 0.15–0.36). Most values of the C:N ratio fell within the expected “acceptable range” of 8–30 for the agricultural soils. As expected, the topsoil (0–0.10 m) generally exhibited lower C:N ratios, reflecting the higher nitrogen content in surface horizons, while higher C:N ratios were observed in some deeper layers (Fig. 4). Part of this variability may also relate to the within-season phase of pasture or crop growth at the time of sampling, as C:N ratios can shift with seasonal changes in plant detritus—such as senesced leaves, stubble, or dead roots—which differ in both carbon and nitrogen content.

### Carbon fractions

Mass balance and analytical consistency were used to validate the SOC fractionation data. Gravimetric recoveries of size fractions were typically >98%, and carbon mass recoveries between 85–115% were accepted, with samples outside this range reprocessed. The final dataset was screened for non-physical values and checked for internal consistency between coarse and fine fractions and whole-soil SOC.

### Permanganate oxidizable carbon

**Fig. 5:**
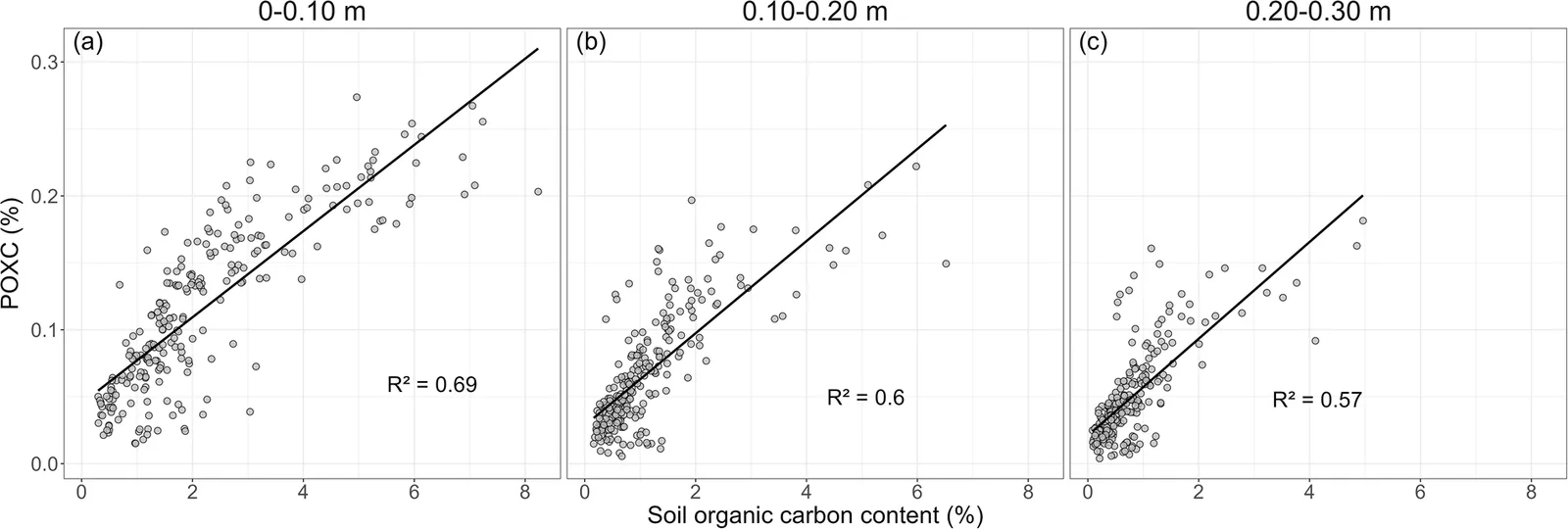
Variation of measured permanganate oxidizable carbon POXC to total carbon across three depths.

POXC data were first screened for implausible values (negative concentrations, POXC exceeding total soil C, and extreme POXC:total C ratios), which were removed or flagged as quality control (QC) failures. POXC was positively correlated with total C (Fig. 5), and values consistently decreases with increasing depth.

### EC, pH in water and pH in CaCl_2_

both pH in water and CaCl_2_ values fall in the physical range (0–14) and the realistic range for mineral soils (3–10.5). Where both pH in water and pH in CaCl₂ were available, we checked that the difference between measurements fell within 0.5–1.5 units, consistent with expected electrolyte effects and within profiles, abrupt changes in pH exceeding 2 units between adjacent horizons were also identified for review (none have been found).

EC values < 0 (invalid) or > 1,000 µS cm^−1^ (physically implausible) were excluded. Vertical consistency was assessed by examining EC profiles for abrupt step changes (e.g. > 10-fold differences between adjacent depths) that were not supported by accompanying information on soil type or known salinity.

### Particle size distribution

Records of clay, silt, and sand fractions with missing values, non-physical values (fractions < 0 or > 100%), or particle size sums outside 95–105% were flagged and excluded. Internal consistency was assessed by verifying that the sum of clay, silt, and sand approximated 100% for all retained samples.

### General management

The data reflect the fact that most of Australia agriculture production is rainfed, consistent with the current literature, with cotton and poppy production as exceptions. The overall trend of the sites in the survey were consistent with Australian literature where after 2010 most of the dryland cropping has moved towards no tillage, and decreased use of stubble burning and increased use of stubble retention and stubble grazing^30^. Overall, our observations were in accordance with more extensive and larger surveys (from the Grain Research and Development Cooperation Paddock Practice Survey^31^). Fertilizer application rates were aggregated by crop type, due to their strong crop-specific dependency. Average nitrogen fertilization rates have increased significantly for most crops, while still remaining below global averages (~150 kg N ha^−1^ for cereal, and oilseed, and maize^32^). In contrast, phosphorus and potassium inputs have remained relatively stable.

### Yield

**Fig. 6:**
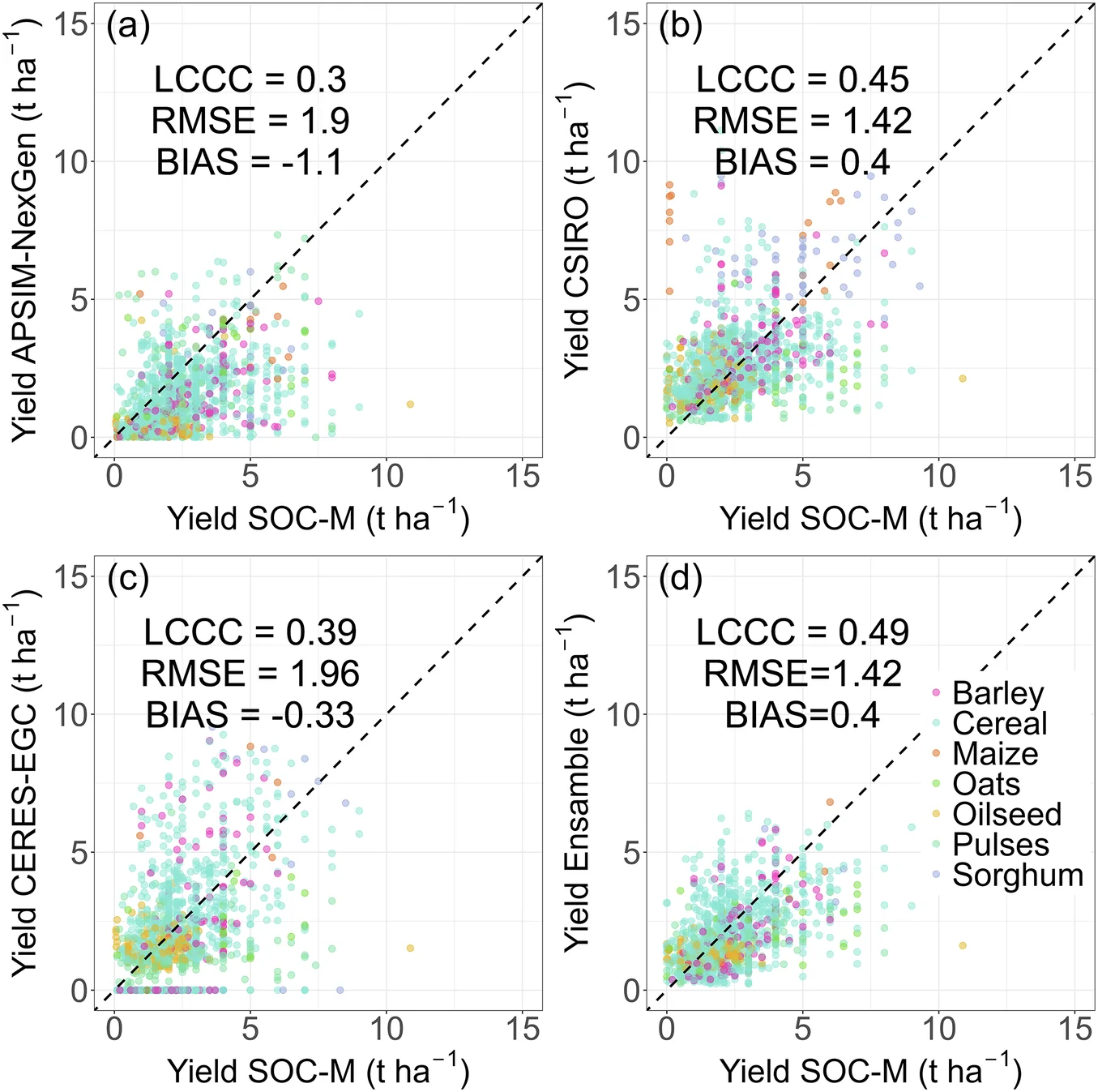
Yield estimation using: (**a**) APSIM-NexGen, (**b**) CSIRO model, (**c**), CERES_EGC, (**d**) taking the average of the three model.

The three models differ in how they represent crop growth and sources of uncertainty. APSIM-NextGen and CERES-EGC simulate yields through detailed crop–soil–weather interactions, while the simpler CSIRO empirical model estimates potential yield mainly from seasonal water availability. Validation at the data-rich site shows that the empirical model performs reasonably well (LCCC = 0.39; RMSE = 1.74 t ha^−1^; bias = 0.12), with predictions clustering around the 1:1 line (Fig. 6). The process-based models capture key trends but show slightly lower accuracy and greater variability (APSIM: LCCC = 0.29, RMSE = 1.93, bias = 1.16; CERES-EGC: LCCC = 0.39, RMSE = 1.96, bias = –0.33). However, APSIM-NextGen better identifies temporal trends, outperforming the other models in detecting monotonic changes using the Mann–Kendall test (Figure S5).

To improve robustness, we averaged predictions from all three models. This ensemble approach leverages complementary strengths, reduces individual model biases, and mitigates structural uncertainty, particularly where calibration data are sparse^16^. The ensemble produces more stable yield estimates with consistent spatial and temporal patterns, though some magnitude differences remain due to model-specific assumptions (e.g., planting density, sowing date, nitrogen responses, phenology).

### Carbon input

**Fig. 7:**
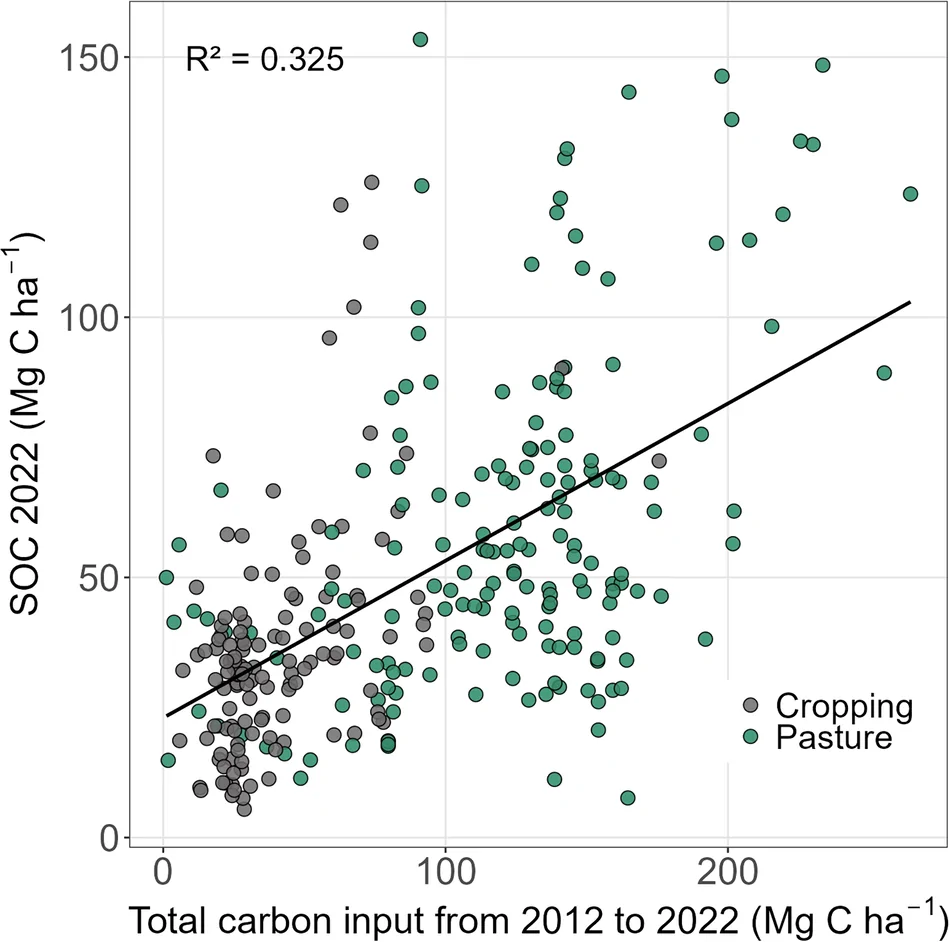
Cumulative carbon input between the two soil surveys against soil organic carbon stocks. The black line represents the general linear trend.

Direct measurements to independently validate estimated carbon inputs were not available. As a proxy, we compared the cumulative carbon inputs between the two sampling periods with total soil organic carbon stocks in the top 0–0.30 m, calculated as the sum along the soil profile of concentration × depth increment × bulk density × (1 – gravel fraction). Carbon stocks were not adjusted to an equivalent soil mass basis in this instance, as no direct temporal comparison of stocks is undertaken in analysis. In line with theoretical expectations, sites with greater estimated carbon inputs generally had higher SOC stocks (Fig. 7). To further examine system-level differences, we stratified the analysis by land use, which confirmed that pasture systems maintained the largest SOC stocks, consistent with long-term observations in Australian agricultural soils^33^.

## Usage notes

The SOC-M dataset provides harmonised information on concentrations and composition of soil organic carbon (SOC), soil properties, and farm management practices across 308 agricultural sites in Australia, covering the period 2000–2023. This resource is particularly valuable for researchers, policymakers, and land managers seeking to understand SOC dynamics under contrasting agricultural systems and management strategies.

The dataset can be used to (i) benchmark SOC stocks and changes across major farming regions, and evaluate differences between management systems (e.g., tillage intensity, stubble handling, grazing); (ii) identify drivers and barriers to SOC change, including climate variability, land-use transitions, and farm management practices; (iii) support SOC modelling and MRV frameworks by providing empirical benchmarks for crop- and pasture-derived carbon inputs, and by enabling robust testing of model assumptions for use in carbon accounting and crediting schemes; (iv) assess policy and practice impacts over time, such as the effectiveness of stubble retention incentives, no-till adoption, or rotational grazing in improving soil carbon outcomes; (v) contribute to national and international reporting, for example by informing Australia’s National Greenhouse Gas Inventory or contributing to global carbon stock assessments.

Importantly, SOC-M can be directly linked to the earlier SCaRP through the *SCaRP_ID*. When combined, the two datasets provide paired measurements of SOC and associated metadata approximately a decade apart, offering a unique longitudinal perspective on soil change at the same 308 sites. This linkage enables robust ex-post analyses of SOC trajectories and the benchmarking of carbon input estimates, while also allowing researchers to examine how changes in management practices or climate have influenced SOC over time.

The daily climate records from the SILO database^24^ and 1 km climate database used within the CSIRO simple crop mode (NIR Volume 2 reference here) used in the simulations are not released as part of this dataset, but they can be independently accessed and linked to SOC-M sites using their geographic coordinates. These long-term weather variables enable integration of climate, soil and management histories, and support ex-ante modelling of SOC stocks under different scenarios. The SOC-M dataset is also designed to be fully interoperable with major national open-data resources, including the Soil and Landscape Grid of Australia (SLGA) for soil attributes, ABARES land use and land management layers, and Bureau of Meteorology climate datasets. Linking SOC-M with these complementary resources allows users to situate site-level SOC observations within broader spatial, environmental, and socio-economic contexts, supporting both fine-scale process studies and national-scale assessments.

We recommend that users apply appropriate screening for data quality and outliers, particularly when integrating management records with soil and climate data.

## Supplementary information

Supplementary information

## Data Availability

The survey data are available on CSIRO Data Portal https://doi.org/10.25919/z378-4286^34^.

## Code availability

The codes are available on figshare: https://doi.org/10.6084/m9.figshare.30716204.
