# Supplementary material for: Key soil attributes and land-management dataset for Australian agroecosystems linking baseline and resampling surveys
Source: Sci Data. 2026 Jul 14;13:1527. doi: 10.1038/s41597-026-07850-8 (PMC13737057; doi:10.1038/s41597-026-07850-8)
Supplement: Supplementary file 1 [file 41597_2026_7850_MOESM1_ESM.docx]

Key soil attributes and land‑management dataset for Australian agroecosystems linking baseline and resampling surveys

### Authors

**Chiara Pasut^1^, Christina Asanopoulos ^1^, Xueyu Zhao^2^,** **Mark Farrell ^1^, Ming Li^3^, Andrea Powell^8^, Georgia Reed^2^, Paul Adkins**^1^**, Thomas Carter^1^, Anna McBeath^1^, Janine McGowan^1^, Sheridan Morton^1^, Steve Szarvas^1^, Kumara Weligama^2^, David Beutel^1^, Hue Dang^1^, Sara Ormond^1^, Bonnie Armour** ^4^**, Glenn Brown^5^, Doug Crawford ^6^, Mary Garrard^7^, Frances  Hoyle^8^, Ronny Lauerwald^9^, Raia Silvia Massad^9^, Malcom McCaskill^10^, Massimiliano De Antoni Migliorati ^11^, Rob Moreton ^5^, Tamara  O’Keeffe^8^, Katherine Polain ^7^, Steven Reeves ^11^, Amanda Schapel ^4^, Liz Sower^12^, Brian Wilson^7^**, **Senani Karunaratne^2^**

Corresponding author(s): Senani Karunaratne ([Senani.Karunaratne@csiro.au](mailto:Senani.Karunaratne@csiro.au)) and Chiara Pasut ([chiara.pasut@csiro.au](mailto:chiara.pasut@csiro.au))

# Sample preparation workflow: from field to laboratory

Figure S1.Initial workflow for sample preparation

Figure S2. Analysis pipeline for preparing <2 mm soil samples to obtain a homogenous representative sample for further analyses, based upon the 2021 Soil Carbon Method.

Figure S3. Workflow to calculate the gravimetric water content and back calculation of oven dried fine and whole soil weight of the collated soil sample based on 2021 Soil Carbon Method.

Figure S4. Pipeline of the particle size fractionation procedure and soil organic matter characterisation using ^13^Carbon nuclear resonance (NMR).

Figure S5. Kendal coefficient to look at the monotonic time trend performance of (a) APSIM, (b) CERES_EGC, (c) CSIRO model, (d) ensemble average of the three models.

# Data simplification and harmonization

Table S 1. Tillage practices reclassification

| **SOCM tillage** | **Simplify label** |
| --- | --- |
| Chisel plough, Mouldboard, spading,  Scarifier (occur before sowing and after harvest > 2 working ) | conventional- >2 working |
| Winged tynes, knife points, topsoil slotting plate (one working plus sowing) | 1 working+sowing |
| Knife points, blade, No till, winged tynes,  knife points, topsoil slotting plate, blade, planter, Tine, Disc seeder | zero till-only sowing |
| Deep ripping | Deep ripping |

Table S 2.Crop parameters used in APSIM-NexGen and CERES-EGC

| Crop ID | Model crop ID | Extra note | Cultivar | Plant population (/m^2^ | Sowing Depth (mm) | Row spacing (mm) |
| --- | --- | --- | --- | --- | --- | --- |
| Cereal | Wheat |  | Hartog | 250 | 30 | 250 |
|  |  | CESER |  | 250 | 30 |  |
| Grain legume | Soybean |  | Generic_MG0 | 50 | 30 | 400 |
|  |  | CERES |  | 50 | 30 |  |
| Oilseed | Canola |  | GenericWinter | 45 | 25 | 180 |
|  |  | CERES |  | 55 | 40 |  |
| Cereal hay | Oats |  | Wintaroo | 120 | 30 | 250 |
|  |  | CERES |  | 120 | 30 |  |
| Annual/Perennial | Red Clover |  | Colenso | 500 | 5 | 150 |
| Maize | Maize | Rainfed system | Dekalb_XL82 | 3 | 30 | 350 |
|  |  | Irrigated system | Pioneer_33M54 | 9 | 30 | 350 |
|  |  | CERES |  | 6 | 30 |  |
| Barley | Barley |  | Dash | 120 | 30 | 750 |
|  |  | CERES |  | 120 | 30 |  |
| Chickpea | Chickpea |  | Striker | 30 | 30 | 450 |
|  |  |  |  | 30 | 30 |  |
| Sorghum | Sorghum |  | N_14NUS04 | 35 | 30 | 750 |
|  |  | CERES |  | 35 | 30 |  |
|  |  |  |  |  |  |  |

Table S 3. Climate and soil properties

| **Variable** | **APSIM-NexGen** | **CERES-EGC** | **CSIRO model** | **Source** |
| --- | --- | --- | --- | --- |
| **Dynamic variables** | | | | |
| Rainfall | daily | daily | daily | (Jeffrey et al., 2001) |
| Temperature | Min & max daily | Min & max daily | daily | (Jeffrey et al., 2001) |
| Solar radiation | daily | daily |  | (Jeffrey et al., 2001) |
| Pan evapotranspiration | daily | daily | daily | (Jeffrey et al., 2001) |
| Vapour pressure | daily |  |  | (Jeffrey et al., 2001) |
| **Soil variables** | | | | |
| Clay, sand, silt | clay, sand, silt | clay, sand, silt | clay |  |
| Bulk density |  |  |  | (Malone et al., 2025) |
| plant available water content |  |  |  | (Malone et al., 2025) |
| Inorganic nitrogen pools (NO_3_, NH_4_, Urea) |  |  |  |  |
| pH |  |  |  | (Malone et al., 2025) |
| drainage limit |  |  |  | (Malone et al., 2025) |
| field capacity |  |  |  | (Malone et al., 2025) |
| Initial soil water content |  |  |  | (Johnston et al., 2003) |
| hydraulic conductivity |  |  |  |  |
| Carbon, CN ratio, Fractions |  |  |  | (Baldock et al., 2013) |

# Crop model simulation set up: more detailed

Figure S 6. CERES-EGC calibration output

Figure S 7. Model intercomparison performance by crop type

Table S 4. Crop model intercomparison performance.

| Crop Type | Model | LCC**C** | RMSE(t ha^-1^) | BIAS |
| --- | --- | --- | --- | --- |
| Oilseed | **APSIM-NexGen** | **0.14** | **1.55** | **1.14** |
|  | **CSIRO** | **0.10** | **1.24** | **-0.42** |
|  | **CERES-EGC** | **0.01** | **1.18** | **0.17** |
|  | **Average** | **0.14** | **1.05** | **0.30** |
| Cereal | **APSIM-NexGen** | **0.33** | **1.82** | **1.03** |
|  | **CSIRO** | **0.34** | **1.58** | **-0.20** |
|  | **CERES-EGC** | **0.44** | **1.97** | **0.13** |
|  | **Average** | **0.46** | **1.45** | **0.32** |
| Pulses | **APSIM-NexGen** | **0.43** | **1.77** | **0.88** |
|  | **CSIRO** | **0.27** | **1.53** | **0.51** |
|  | **CERES-EGC** | **0.18** | **1.72** | **0.61** |
|  | **Average** | **0.46** | **1.47** | **0.67** |
| Barley | **APSIM-NexGen** | **0.44** | **1.81** | **1.39** |
|  | **CSIRO** | **0.48** | **1.18** | **-0.02** |
|  | **CERES-EGC** | **0.48** | **2.22** | **0.32** |
|  | **Average** | **0.55** | **1.32** | **0.57** |
| Oats | **APSIM-NexGen** | **0.43** | **2.26** | **1.15** |
|  | **CSIRO** | **0.30** | **2.28** | **0.97** |
|  | **CERES-EGC** | **0.5** | **2.61** | **1.88** |
|  | **Average** | **0.54** | **2.19** | **1.34** |
| MAIZE | **APSIM-NexGen** | **0.09** | **2.42** | **2.32** |
|  | **CSIRO** | **0.14** | **1.83** | **-1.02** |
|  | **CERES-EGC** | **0.03** | **1.29** | **-0.27** |
|  | **Average** | **0.15** | **1.21** | **0.34** |
| sORGHUM | **APSIM-NexGen** | **0.06** | **2.16** | **1.04** |
|  | **CSIRO** | **0.24** | **3.42** | **-2.25** |
|  | **CERES-EGC** | **0.10** | **4.10** | **1.98** |
|  | **Average** | **0.30** | **1.91** | **0.26** |
| All crop | **APSIM-NexGen** | **0.28** | **1.93** | **-1.16** |
|  | **CSIRO** | **0.39** | **1.74** | **0.12** |
|  | **CERES-EGC** | **0.39** | **1.96** | **-0.33** |
|  | **Average** | **0.48** | **1.42** | **0.41** |

Table S 5.Crop parameters table with yield components, allocation of dry matter and associated carbon contents for each plan component (based on Baldock et al., 2025).

| Crop  Type  *Crop Type* | Yield Component  *YieldComp* | Carbon content of each plant component  (g C/ g plant component dry matter) | | | | | Allocation of plant total dry matter to plant components  (g plant component dry matter/100 g total plant dry matter) | | | | |
| --- | --- | --- | --- | --- | --- | --- | --- | --- | --- | --- | --- |
|  |  | Grain  *CFracGbfrA* | Stalk  *CFracStlkA* | Leaf  *CFracLeafA* | Fine Root  *CFracCortA* | Coarse Root  *CFracFirtA* | Stalk  *allocStlkA* | Grain  *allocGbfrA* | Leaf  *allocLeafA* | Coarse Root  *allocCortA* | Fine Root  *allocFirtA* |
| Mung bean | grain | 0.434 | 0.45 | 0.375 | 0.45 | 0.375 | 0 | 23 | 54 | 0 | 23 |
| Narrow-leaf lupin | grain | 0.45 | 0.45 | 0.3877 | 0.45 | 0.3877 | 0 | 22 | 55 | 0 | 23 |
| Oat | grain | 0.4343 | 0.45 | 0.4375 | 0.45 | 0.441 | 0 | 16 | 61 | 0 | 23 |
| Oil poppies | grain | 0.4343 | 0.45 | 0.4375 | 0.45 | 0.441 | 0 | 38.5 | 38.5 | 0 | 23 |
| Peanut | grain | 0.53 | 0.45 | 0.42 | 0.45 | 0.41 | 0 | 25 | 52 | 0 | 23 |
| Rice | grain | 0.4343 | 0.45 | 0.4375 | 0.45 | 0.441 | 0 | 31 | 46 | 0 | 23 |
| Sorghum | grain | 0.4343 | 0.45 | 0.4375 | 0.45 | 0.4375 | 0 | 35.2 | 41.8 | 0 | 23 |
| Soybean | grain | 0.434 | 0.45 | 0.375 | 0.45 | 0.375 | 0 | 23 | 54 | 0 | 23 |
| Sugarcane | stalks | 0.45 | 0.39 | 0.441 | 0.45 | 0.415 | 64 | 0 | 13 | 0 | 23 |
| Sunflower | grain | 0.45 | 0.45 | 0.45 | 0.45 | 0.45 | 31 | 31 | 15 | 0 | 23 |
| Triticale | grain | 0.4343 | 0.45 | 0.4375 | 0.45 | 0.441 | 0 | 26 | 51 | 0 | 23 |
| Vetch | grain | 0.434 | 0.45 | 0.375 | 0.45 | 0.375 | 0 | 30 | 47 | 0 | 23 |
| Wheat | grain | 0.4343 | 0.45 | 0.4375 | 0.45 | 0.441 | 0 | 27.5 | 49.5 | 0 | 23 |
| Lucerne | leaves | 0.45 | 0.45 | 0.4067 | 0.45 | 0.4067 | 0 | 0 | 53 | 0 | 47 |
| Perennial grass | leaves | 0.45 | 0.45 | 0.409 | 0.45 | 0.409 | 0 | 0 | 53 | 0 | 47 |

# References

Baldock, J. A., Hawke, B., Sanderman, J., & Macdonald, L. M. (2013). Predicting contents of carbon and its component fractions in Australian soils from diffuse reflectance mid-infrared spectra. *Soil Research*, *51*(8), 577. https://doi.org/10.1071/SR13077

Jeffrey, S. J., Carter, J. O., Moodie, K. B., & Beswick, A. R. (2001). Using spatial interpolation to construct a comprehensive archive of Australian climate data. *Environmental Modelling & Software*, *16*(4), 309–330. https://doi.org/10.1016/S1364-8152(01)00008-1

Johnston, R. M., Barry, S. J., Bleys, E., Bui, E. N., Moran, C. J., Simon, D. a. P., Carlile, P., McKenzie, N. J., Henderson, B. L., Chapman, G., Imhoff, M., Maschmedt, D., Howe, D., Grose, C., Schoknecht, N., Powell, B., & Grundy, M. (2003). ASRIS: The database. *Soil Research*, *41*(6), 1021–1036. https://doi.org/10.1071/sr02033

Malone, B. P., Searle, R., Stenson, M., McJannet, D., Zund, P., Román Dobarco, M., Wadoux, A. M. J.-C., Minasny, B., McBratney, A., & Grundy, M. (2025). Update and expansion of the soil and landscape grid of Australia. *Geoderma*, *455*, 117226. https://doi.org/10.1016/j.geoderma.2025.117226

Richetti, J., Lawes, R. A., Whan, A., Gaydon, D. S., & Thorburn, P. J. (2024). How well does APSIM NextGen simulate wheat yields across Australia using gridded input data? Validating Continental-Scale Crop Model Simulations. *European Journal of Agronomy*, *158*, 127212. https://doi.org/10.1016/j.eja.2024.127212

Sacks, W. J., Deryng, D., Foley, J. A., & Ramankutty, N. (2010). Crop planting dates: An analysis of global patterns. *Global Ecology and Biogeography*, *19*(5), 607–620. https://doi.org/10.1111/j.1466-8238.2010.00551.x

Unkovich, M., Baldock, J., Marvanek, S., Unkovich, M., Baldock, J., & Marvanek, S. (2009). Which crops should be included in a carbon accounting system for Australian agriculture? *Crop and Pasture Science*, *60*(7), 617–626. https://doi.org/10.1071/CP08428

Verburg, K., Bond, W. J., & Hunt, J. R. (2012). Fallow management in dryland agriculture: Explaining soil water accumulation using a pulse paradigm. *Field Crops Research*, *130*, 68–79. https://doi.org/10.1016/j.fcr.2012.02.016
